# First Survey on the Seroprevalence of *Coxiella burnetii* in Positive Human Patients from 2015 to 2024 in Sardinia, Italy

**DOI:** 10.3390/pathogens14080790

**Published:** 2025-08-07

**Authors:** Cinzia Santucciu, Maria Paola Giordo, Antonio Tanda, Giovanna Chessa, Matilde Senes, Gabriella Masu, Giovanna Masala, Valentina Chisu

**Affiliations:** Zoonotic Diseases-WOAH and NRL for Echinococcosis, Animal Health, Istituto Zooprofilattico Sperimentale Della Sardegna, 07100 Sassari, Italy; mariapaola.giordo@izs-sardegna.it (M.P.G.); antonio.tanda@izs-sardegna.it (A.T.); giovanna.chessa@izs-sardegna.it (G.C.); matilde.senes@izs-sardegna.it (M.S.); gabriella.masu@izs-sardegna.it (G.M.); giovanna.masala@izs-sardegna.it (G.M.)

**Keywords:** *Coxiella burnetii*, Q fever, zoonotic pathogens, Sardinian pathogens, human diagnosis

## Abstract

*Coxiella burnetii*, the etiological agent of Q fever, is a globally distributed zoonotic pathogen affecting both animals and humans. Despite its known endemicity in various Mediterranean regions, data on human seroprevalence in Sardinia are still lacking. This study aimed to assess seroprevalence in patients and to analyze the annual positivity rate related to the serum samples collected in Sardinia over a ten-year period (2015–2024). For this purpose, a total of 1792 patients were involved in the survey, and 4310 serum samples were analyzed using indirect immunofluorescence assay (IFI) to detect IgM and IgG antibodies against *C. burnetii*. The global seroprevalence rates relating to all the patients over a ten-year period were determined along with the annual positivity rate and trends from all sera. An overall seroprevalence of 27.0% and an average of annual positivity rate of 16.0% were determined, with the IFI detecting IgG antibodies in 15.2% of positive samples and IgM antibodies in 0.9%, suggesting significant prior exposure of the population evaluated. Annual positivity rates ranged from 24.8% in 2016 to 8.0% in 2020. These results confirmed the endemic circulation of *C. burnetii* in Sardinia and the ongoing risk of human exposure. A GIS-based map was built to evidence the spatial distribution of Q fever in Sardinia. Interestingly, areas with higher seroprevalence appear to coincide with the distribution of sheep and goat farms, indicating a link between livestock and human exposure. These findings confirm the circulation of *C. burnetii* in Sardinia and underscore the importance of epidemiological monitoring, public health interventions, and educational efforts in populations at increased risk.

## 1. Introduction

*Coxiella burnetii* (family *Coxiellaceae*, order *Legionellales*, class *Gammaproteobacteria*) is a Gram-negative bacterium responsible for coxiellosis in animals and Q fever in humans, a globally distributed zoonosis [1]. Although traditionally classified as an obligate intracellular pathogen, *C. burnetii* can now be cultivated under axenic conditions using specialized cell-free media that mimic the intracellular environment [2,3]. This pathogen exhibits a biphasic developmental cycle, alternating between two morphological forms: the large cell variant (LCV), which is metabolically active and replicative, and the small cell variant (SCV), a spore-like, dormant, and environmentally resistant form. *C. burnetii* was first described in 1937 in Australia by Edward Derrick, following an outbreak of febrile illness among slaughterhouse workers [4]. Since then, with only the exception of New Zealand, *C. burnetii* has been reported worldwide in a wide range of animal species.

Humans and animals can become infected with *C. burnetii* by several transmission routes. The primary mode of human infection is the inhalation of contaminated aerosols due to environmental contamination, including from soil and dust particles [5]. The high bacterial load shed during parturition, especially from birth products, urine, feces, and milk from infected animals, and the environmental persistence of the SCV form of *C. burnetii,* play a critical role in transmission dynamics. Although less common, transmission can also occur through the consumption of unpasteurized dairy products or tick bites [5,6,7]. More rarely, direct contagion between humans or from animals to humans has been reported [8]. A wide range of animal species, particularly domestic ruminants, act as reservoirs, representing a significant zoonotic threat. Wild animals may also contribute to the environmental dissemination of the pathogen [9]. Additionally, sexual transmission has been experimentally demonstrated, since viable *C. burnetii* has been found in semen [10].

*C. burnetii* is classified as a biological agent of group III. Workers involved in the management of this bacterium must adhere to strict biosafety guidelines, since Q fever is widely recognized as an occupational hazard for high-risk groups, such as livestock farmers and slaughterhouse workers. Because laboratory technicians are more likely to be infected when handling clinical samples from suspected subjects, these have to be handled with caution to avoid any contamination of either personnel or workplaces, including from any other pathological agent.

Clinical symptoms of acute Q fever are not pathognomonic and may be easily confused with those of other disorders. Patients mostly manifest three clinical forms, flu-like illness, pneumonia, and hepatitis [10,11,12]. Chronic Q fever is instead frequently characterized by endocarditis or vascular infection along with other forms [13]. In animals, coxiellosis can cause severe symptoms, including high rates of abortion [5], leading to economic losses and low milk production [14].

Experienced clinicians can correctly identify suspected human cases that may be addressed with appropriate medical management, but only laboratory investigations are usually conclusive. Diagnosis of Q fever in humans is mainly based on serological tests for the detection of IgG and IgM antibodies against *C. burnetii*. The distinction between antibodies directed against antigens of phase I and phase II of *C. burnetii* is also required to differentiate acute from chronic Q fever. The indirect immunofluorescent assay (IFI) is considered as the gold standard for serological diagnosis due to its high sensitivity and specificity. Other tests, such as the enzyme-linked immunosorbent assay (ELISA), Western blot (WB), and the complement fixation test (CFT), are also used, but less frequently and often as supplementary assays. The diagnosis of acute Q fever is based on seroconversion or a four-fold rise in IgG and IgM antibody titers between two sera collected at a distance of 3–6 weeks [15].

Direct detection methods include analysis based on polymerase chain reaction (PCR), most commonly targeting different genes. In addition to serology and PCR, reference laboratories may perform in vitro *C. burnetii* cultures in order to avoid any false positives and to provide information on the vitality of the pathogen. In recent years, next-generation sequencing (NGS) has emerged as a powerful tool for rapid screening, simultaneous identification, and genotyping of clinical samples [16].

This study aimed to assess the seroprevalence of *C. burnetii* infection in human patients from Sardinia (Italy) and the annual positivity rate related to the serum samples collected over a ten-year period (2015–2024). To our knowledge, this is the first report focusing on the seroprevalence of Q fever on the island.

In this survey, the serological study was conducted on samples collected from patients with suspected infection who underwent laboratory investigations. The percentage of positive cases was determined and their geographical distribution was represented as a GIS map. The unique environmental conditions of the island have facilitated the circulation of several zoonotic pathogens, including *C. burnetii* [16,17]. These results provide valuable insight into the regional burden of Q fever, help identify at-risk populations, and support strategies for disease prevention and control.

## 2. Materials and Methods

### 2.1. Study Area

**Figure 1:**
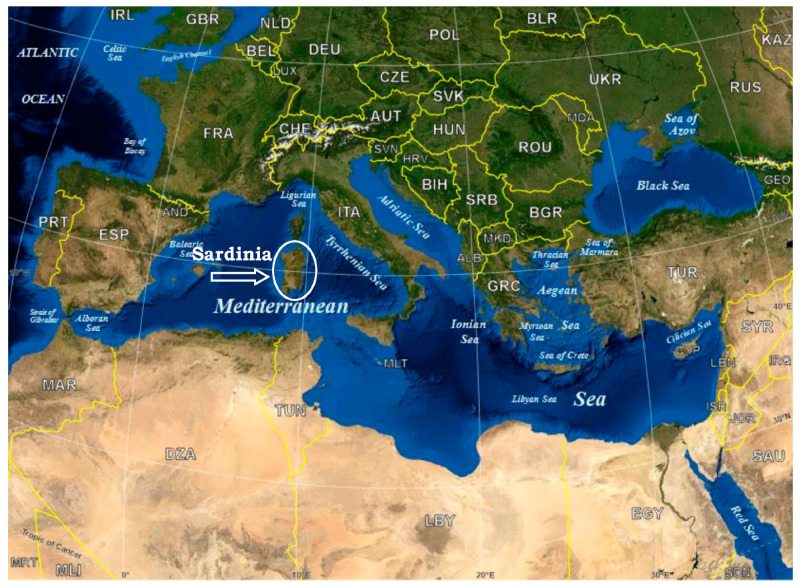
Map of Sardinia. Source: https://commons.wikimedia.org/wiki/File:Mediterranean_Sea_political_map-en.svg (accessed on 3 August 2025).

At the time of the study all the patients involved in this survey lived in Sardinia, the second-largest Italian island after Sicily, located in the middle of the Mediterranean Sea (Figure 1). In detail, with an area of 24,100 km^2^, Sardinia is located between 38°51′ and 41°18′ latitude north and 8°8′ and 9°50′ longitude east, south of Corsica and west of Spain, with mainland Italy to the east and North Africa to the south. Sardinia is known for its diversified landscape, featuring mountains, flat areas, rocky coastlines, and some of the most pristine beaches in Europe. For these reasons, Sardinia has been considered as a microcontinent. Significant seasonal variations have been described, with temperate conditions in the middle seasons, hot and dry summers, and two distinct climatic zones: cold mountainous areas and warm coastal regions. Its central position has made it a historical crossroads over the centuries, and given its insulation and peculiar characteristics, high biodiversity occurs on this island, which has favored the spread of zoonotic agents in the Sardinian territory.

Moreover, the population of Sardinia, defined on the basis of the most recent census as of 31 December 2023, amounts to 1,570,453 residents [(Censimento-permanente-popolazione_Anno-2023_Sardegna.PDF (www.istat.it)]. The number of inhabitants is lower in comparison to that of domestic ruminants. Sardinia has a highly dense ovine population, about 2.5 million, according to the National Italian Database (BDN), established by the Ministry of Health at the National Surveillance Centre of the Istituto Zooprofilattico of the Abruzzo and Molise region. Sheep are often managed in intensive conditions; seasonal farming practices and the semi-arid climate may favor the environmental persistence of the bacterium.

### 2.2. Sampling

A total of 1792 patients were involved in the study during the period ranging from 2015 to 2024. They were referred to physicians or hospitals located in the north of Sardinia, and their blood samples were collected and sent to the laboratories of Istituto Zooprofilattico Sperimentale della Sardegna (IZS) upon medical request. Patients’ general data, diagnostic tests, and results were recorded in a database. The retrospective survey analyses of the records showed a total of 4310 tests performed on samples from the patients suspected of having Q fever. In detail, serum samples were serologically examined for detection of anti-*Coxiella* antibody, respectively 2230 for IgM and 2080 for IgG. All tests were carried out on all specimens from the patients involved in the study, including those subjected to a single clinical examination as well as those followed up; consequently, in these patients, blood samples were collected several times, mostly over a wide period of years, and submitted to analyses for *C. burnetii* detection.

### 2.3. Laboratory Investigations

#### Indirect Immunofluorescence

A total of 4310 blood samples, collected without anticoagulant from 1792 patients involved in this study, were examined by an indirect immunofluorescence assay (IFI) to detect antibodies against *C. burnetii*. After clot formation, the samples were centrifuged at 350 g (2500 rpm) for 5 min in order to obtain clear sera. In detail, 2080 sera were tested for IgG antibodies and 2230 for IgM antibodies.

The IFI assay used commercial slides coated with *C. burnetii* antigen (Fuller Laboratories, Fullerton, CA, USA). An incubation step in a humid and dark chamber was performed at a temperature of 37 °C ± 2 for 30 min with 10 μL of each serum to be examined. If present, the antibodies against *C. burnetii* were detected by adding 10 μL of anti-human-IgG or anti-IgM labeled with Fluorescein Isothiocyanate (FITC). The slides were then washed with 1X phosphate-buffered saline (PBS). Fluorescence signals indicating positive antigen–antibody reactions were observed under a fluorescence microscope. After each step, serum-positive and -negative field controls were also included in each analysis to ensure test validity.

Regarding diagnostic criteria, IgM and IgG anti-*C. burnetii* antibodies with titers ≥ 1:50 were considered as the cutoff to define a positive serological result, and this indicated exposure to the pathogen. Due to the absence of phase-specific serology (IgG phase I vs. phase II) and clinical data, it was not possible to distinguish between chronic and acute infection.

### 2.4. GIS-Based Survey of Patients’ Positive Distribution Sites

The Geographic Information System (GIS) coordinates of the origins of the subjects who tested positive for *C. burnetii* by serological tests were also detected and recorded. The GIS-based surveys were conducted from 2015 to 2024. For this purpose, all geographical coordinates were detected and managed with QGIS software (version 3.38.2). The corresponding sites were used to construct a map to evidence the spatial distribution of Q fever in Sardinia.

### 2.5. Seroprevalence Evaluation

The global seroprevalence of *C. burnetii* infection in the period of 10 years covered in this study (2015–2024) was assessed by using the following formula:

$$Seroprevalence=\frac{Number\;of\;Q\;fever\;positive\;patients}{Total\;number\;of\;patients\;surveyed}$$

The annual positivity rate of *C. burnetii* was assessed for each year in the study (2015 to 2024) by the proportion of serum samples testing positive among all samples analyzed from patients with suspected Q fever:

$$Annual\;positivity\;rate=\frac{Number\;of\;Q\;fever\;positive\;serum\;samples}{Total\;number\;of\;serum\;samples\;surveyed}$$

The mean annual prevalence and standard deviation were also detected. The data in the report were reported yearly in the range considered.

Moreover, the percentage of *C. burnetii*-positive individuals relative to the total population of Sardinia was also detected, by the following formula:

$$Population\;based\;prevalence=\frac{Number\;of\;C.\;burnetii\;positive\;patients}{Total\;of\;Sardinianin\;habitants}$$

## 3. Results

### 3.1. Laboratory Investigations

Laboratory investigations at the individual level revealed that the total number of positive patients was 480; in detail, 448 patients tested positive for anti-Coxiella IgG antibodies, and 32 patients tested positive for IgM.

The analyses of all blood sera included in this study (n = 4310) (Table 1), from patients suspected of having Q fever and investigated in this survey (2015 to 2024), evidenced an annual positivity rate corresponding to 16.0%, matching with n = 694 samples from patients positive for *C. burnetii*. The number of positive samples corresponded to the total number of the blood samples analyzed from each individual, rather than to the number of sera from patients subjected to follow-up.

Details of the findings (Table 1) revealed that, out of all the sera examined by IFI for anti-*Coxiella* antibody detection, n = 38 (0.9%) displayed the presence of IgM, while n = 656 (15.2%) showed IgG antibodies.

### 3.2. GIS-Based Survey of Patients’ Positive Distribution Sites

A GIS-based survey conducted from 2015 to 2024 showed the origins of all the patients positive for *C. burnetii*, corresponding to n = 480.

**Figure 2:**
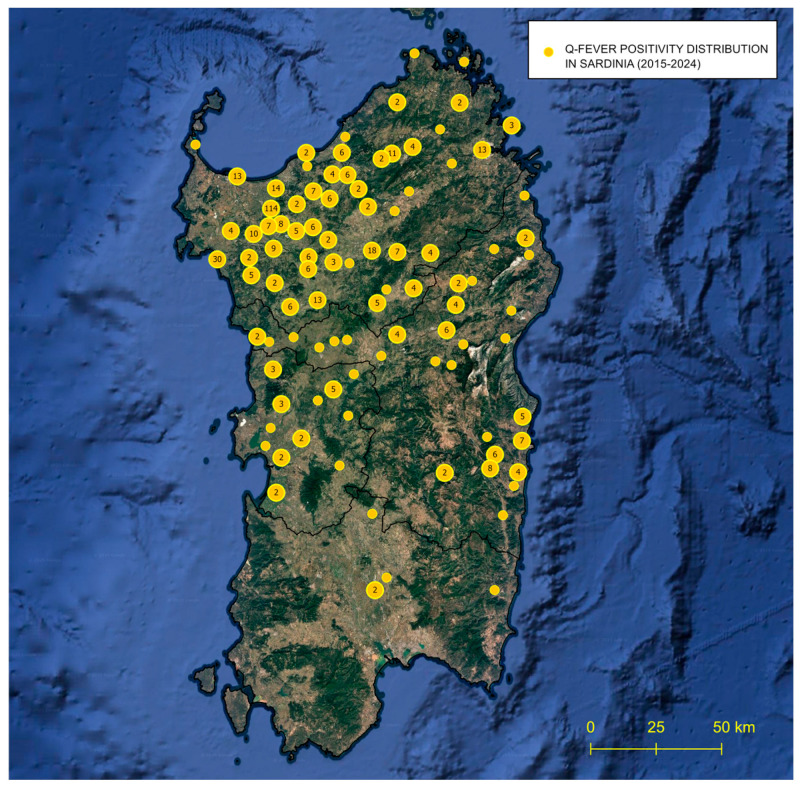
Q fever patients’ distribution in Sardinian territories: smallest spots indicate one positive case, bigger spots show more than one patient, and the number inside the dots specifies the exact number of patients in this spot.

In detail, the results showed that these patients were distributed in all Sardinian territory, excluding mountains and the south (Figure 2), since only patients that were referred to clinical centers in north Sardinia were involved in this study. As displayed on the map, the ocher spots are indicative of the sites with the presence of Q fever.

### 3.3. Seroprevalence Evaluation

Considering the number of Q fever-positive patients was n = 480, the global seroprevalence estimated for infection with *C. burnetii* in the period of 10 years (2015–2024) was 27.0%., determined on the total number of patients, n = 1792.

**Chart 1:**
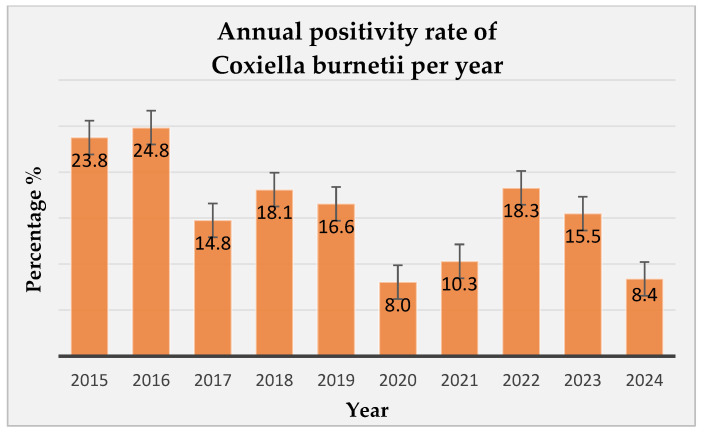
Representation of the percentages for the annual positivity rate for *Coxiella burnetii* in all samples per year.

The determination of the annual positivity rate for *C. burnetii* for all sera examined per year is reported in Chart 1. As displayed, a variable range was detected, with the highest rate of 24.8% in 2016 and the lowest rate of 8.0% in 2020; the values were different for each year, with an average of 16.0% ± a standard deviation of 5.8.

Finally, the percentage of representativeness of Q fever, corresponding to the number of positive patients relative to the general population of Sardinia, was 0.03%.

## 4. Discussion

To the best of our knowledge, this study represents the first report on the seroprevalence of antibodies against *C. burnetii* in the human population of Sardinia. The survey ranged over a ten-year period from 2015 to 2024.

Given the unique environmental features of Sardinia, this region provides an ideal ecological landscape for the persistence and transmission of numerous zoonotic agents, many of which have been extensively documented [5,17,18,19,20]. The Mediterranean climate of Sardinia, extensive livestock farming, and close human-animal interactions, particularly in rural areas, create favorable conditions for zoonotic pathogen maintenance and spillover. Moreover, its high resistance to environmental stresses (including desiccation, UV radiation, atmospheric agents, and temperature extremes) allows this bacterium to survive for prolonged periods in dust, soil, and aerosols. This aspect makes it one of the most environmentally stable and transmissible zoonotic bacteria [21]. In addition, the high seroprevalence of *C. burnetii* in domestic ruminants, particularly sheep, which are abundant in Sardinia, reinforces the status of the island as a natural reservoir for this pathogen [5]. Sardinia has one of the highest sheep densities in Europe, with almost twice as many sheep as inhabitants, with millions of sheep and goats often raised in extensive, open-air systems.

Our findings confirm the notable endemic presence of *C. burnetii* in humans in Sardinia, since a notable endemic diffusion of *C. burnetii* in humans in Sardinia, consistent with previous reports in various animal species, is described. In this study, a global seroprevalence of 27.0% was assessed from all patients investigated (n = 1792) and an annual positivity rate of 16.0% was detected in human blood samples (n= 4310), suggesting widespread exposure to the pathogen among the Sardinian population.

Previous epidemiological studies in Sardinia have reported higher seroprevalence rates of *C. burnetii* in small ruminants, with 38% in sheep and 47% in goats based on ELISA testing of over 9000 sera [5]. As expected, animals usually present a higher prevalence with respect to humans due to the intensive and hygienic conditions of animal breeding and to the fact that only specific categories of workers are typically exposed. Moreover, the discrepancy in seroprevalence between animals and humans could suggest that the disease burden in humans in contact with livestock or contaminated environments may be higher than already reported.

Interestingly, we observed that the seroprevalence distribution of Q fever in humans appears to geographically overlap with the distribution of sheep and goat farms, which is consistent with findings previously reported [22], suggesting a potential key role of sheep in the zoonotic transmission of the infection in Sardinia. Coxiellosis distribution has been reported worldwide, mostly by studies on several animal species [23], on its prevalence in ticks in Europe [7], and across the broader Mediterranean [23,24], Southern Italy [25] and neighbors countries such as Algeria [26]. Although *C. burnetii*-infected animals are often poorly symptomatic, coxiellosis is frequently suspected in livestock due to repeated abortions in the herds. Moreover, in these cases the infection persists and spreads silently because infected animals may often go unnoticed by breeders. These events also represent a critical point of environmental contamination and human exposure risk. With the environmental pressure being very high, this bacterium can be easily transmitted to humans, who could also acquire infection without direct animal contact by inhaling contaminated dust from areas where infected animals have been present [27].

Despite there being several reports of Q fever outbreaks in humans, there is a limited number of studies on the seroprevalence of Q fever. Outbreaks have a high impact on public health, and are usually linked to aerosol exposure, contaminated environments, or contact with livestock, as seen in several outbreaks reported in Spain [28], France [29], and Italy [30]. Similar patterns were observed in countries along the Adriatic coast, including Croatia and Slovenia, where outbreaks have been associated with animal exposure and environmental contamination [31]. Q fever is endemic in many countries across the Mediterranean sea [32], where sporadic cases and multiple outbreaks have been reported over the past decades.

Our data confirmed the trend reported by other surveys, confirming a relevant level of human exposure to *C. burnetii* in Sardinia. The 27.0% seroprevalence rate observed in our study is in line with values reported in other serological surveys across the Mediterranean region. A systematic review and meta-analysis based on 112 studies displays an overall seroprevalence of Q fever of 22.4%, with 25.5% in humans. In animals, the seroprevalence changed depending on species, and the values ranged from 6.3% to 28.1%; in ticks the value was 17.5% [7]. Interestingly, very high human seroprevalence rates have been reported in Greece, Crete, and Cyprus, sometimes exceeding 40% [32,33,34]. In the Maghreb, the Mashreq, and Turkey, Q fever remains an underestimated but persistent zoonotic threat, with several studies reporting substantial seroprevalence rates in humans, especially those in rural or occupational risk groups [35]. In our seroepidemiological study, the findings of the analysis of all samples for Q fever revealed an annual positivity rate of antibodies against C. burnetii of 16.0% ± 5.8% with considerable interannual variation. The yearly positivity rate ranged from the highest value of 24.8% in 2016 to the lowest value of 8.0% in 2020. These fluctuations may reflect multiple factors, including changes in weather patterns, livestock movements, vector population dynamics, and diagnostic tools. Notably, the reduced seroprevalence observed in 2020 (8.0%) may be attributed to the impact of the COVID-19 pandemic, which affected public health surveillance and healthcare. Moreover, in the final period of the investigations, annual positivity rates appeared with a trend that tended toward lower levels. This trend could be interpreted as an improvement in control measures, reduced exposure risks, or potentially a real decline in *C. burnetii* in the environment. However, the plateau may also reflect the possibility of persistent low-level transmission or underdiagnosis due to clinical overlap with other febrile illnesses.

Our results were attributable to the indirect immunofluorescence assay (IFI), with the positivity rate revealed among serum samples being equal to 16.0%, with 15.2% for IgG and only the 0.9% for IgM. The IgG predominance suggests previous exposure in the Sardinian population, likely due to environmental circulation of the pathogen among at-risk occupational groups (e.g., farmers, veterinarians, shepherds). The lower IgM positivity aligns with the hypothesis that acute infections may be underreported or asymptomatic, highlighting the silent and chronic nature of Q fever in endemic areas [36].

In a recent systematic review and meta-analysis, a seroprevalence of 44% was reported among individuals with occupational exposure, particularly veterinarians, abattoir workers, livestock farmers, and agricultural laborers. In contrast, forestry rangers and laboratory workers exhibited either no increase or even reduced risk [37]. This trend contrasts with the national Q fever notification rate in Italy (under 0.1 cases per 100,000 persons annually) and highlights the probable underdiagnosis and underreporting of the disease [38]. Moreover, the percentage of representativeness of Q fever in this survey relative to the general population of Sardinia corresponded to 0.03%.

Our data from the GIS-based survey from 2015 to 2024 evidenced a consistent distribution of *C. burnetii*-seropositive patients (n = 480) along all regional territories, with the exclusion of the south and the mountains (Figure 2). However, this could represent a bias since the subjects involved in this survey were mostly referred to medical centers located in the north of Sardinia and patients often tend to be referred within their districts. An interesting future perspective would be to also evaluate the origins of *C. burnetii*-positive patients from the southern areas.

Another aspect to consider is the potential role of ticks in the transmission of *C. burnetii* [37,38,39]. Although ticks are not recognized as the primary route of infection in humans, they may represent a secondary but relevant transmission pathway, where ecological conditions favor tick survival and proliferation. Due to climate, vegetation, and high density of wild and domestic hosts, Sardinia is a natural hotspot for tick populations and tick-borne pathogens. Recent entomological surveys from the island have highlighted the presence of *C. burnetii* in the local tick fauna [6], as well as the ecological complexity of *Coxiella* spp. within these tick populations [40,41].

A limitation of this study is the inability to differentiate between acute and chronic Q fever, as we were not able to assess phase-specific IgG antibodies. As is known, chronic Q fever is diagnosed with high-IgG phase I titers (>800), which were not evaluated here. The reported seropositivity should be interpreted as a recent or past exposure to *C. burnetii*, and we cannot make any clinical or temporal distinctions. Moreover, these data are derived from patients with symptoms suggestive of Q fever. Therefore, the reported seroprevalence cannot be generalized to the broader asymptomatic population. Additionally, this study involves patients who were clinically suspected of having Q fever and were referred for diagnostic testing. Therefore, the seroprevalence here presented reflects the exposure in this specific cohort and should not be extrapolated to the asymptomatic population of Sardinia. Future investigations should employ random sampling based on the general population to provide a more representative assessment of *C. burnetii* exposure.

The observed decreasing trend and subsequent stabilization of *C. burnetii* seroprevalence over the last five years underscore the importance of continued surveillance and diagnostic vigilance. The data also suggest the need for epidemiological investigations to understand the drivers behind the initial spike and later decline. Further research should explore environmental, occupational exposure, and animal reservoir factors that may influence *C. burnetii* transmission, alongside the role of public health interventions and diagnostic accuracy.

## 5. Conclusions

This study represents the first investigation on seroprevalence of *C. burnetii* in human cases in Sardinia, confirming its diffusion in almost all territories and its endemic nature in the region. It highlights that the unique ecological conditions, extensive livestock farming, and close human-animal interactions of the island contribute significantly to the maintenance and transmission of this zoonotic pathogen. Our findings showed a seroprevalence value of 27.0% determined from the patients (n = 1792) investigated, and an annual positivity rate of 16.0% determined from the sera (n= 4310) examined, confirming the high rate of Q fever in humans in Sardinia, and the average trend of global distribution, as already reported in several animal species. Although the annual positivity rate varied over the years, a notable decrease and stabilization in recent years may reflect improved control measures or changes in exposure risk. These results highlight the importance of continued seroepidemiological surveillance and improved diagnostics, and targeted public health strategies are essential to better understand and control Q fever in Sardinia and similar Mediterranean regions.

## Figures and Tables

**Table 1:** Number and percentage of the total sera investigated from 2015 to 2024 from subjects suspected of having Q fever.

Techniques	n° (%) Positive	n° (%) Negative	n° (%) Total
**IFI-IgG**	656 (15.2)	1424 (33.0)	2080 (48.3)
**IFI-IgM**	38 (0.9)	2192 (50.9)	2230 (51.7)
**Total (%)**	694 (16.0)	3616 (83.9)	4310 (100)

**IFI**: indirect immunofluorescence.

## Data Availability

The data generated during the current study are available from the corresponding authors upon request.
